# Population-based incidence of psoriasis vulgaris in Germany: analysis of national statutory insurance data from 65 million population

**DOI:** 10.1007/s00403-023-02796-y

**Published:** 2024-01-04

**Authors:** Madeline Deike, Jiancong Wang, Ralph Brinks, Stephan Meller, Lennart Ocker, Falk G. Bechara, Jörg H. W. Distler, Xenofon Baraliakos, David Kiefer, Philipp Sewerin

**Affiliations:** 1 Hiller Research Center, University Hospital Düsseldorf, Medical Faculty of Heinrich Heine University, 40225 Düsseldorf, Germany; 2 Institute of Biometry and Epidemiology, The German Diabetes Center, Heinrich Heine University Düsseldorf, Düsseldorf, Germany; 3 Chair for Medical Biometry and Epidemiology, University of Witten/Herdecke, Witten, Germany; 4 Clinic for Dermatology, University Hospital Duesseldorf, Medical Faculty of Heinrich-Heine-University, Duesseldorf, Germany; 5 Department of Dermatology, Venereology and Allergology, St Josef Hospital, Ruhr-University, Bochum, Germany; 6 Clinic for Rheumatology, University Hospital Düsseldorf, Medical Faculty of Heinrich Heine University, Düsseldorf, Germany; 7 Ruhr-Universität Bochum, Rheumazentrum Ruhrgebiet, Herne, Germany

**Keywords:** Psoriasis vulgaris, Population-based, Age-specific, Sex-specific, Age-standardized, Incidence, Germany

## Abstract

Information on the population-based incidence of psoriasis vulgaris was limited. This study was to provide a comprehensive understanding of the age-specific and sex-specific incidence of psoriasis vulgaris in Germany. The data were obtained in the context of a morbidity-based risk adjustment by statutory health insurance companies in Germany, comprising information regarding 65 million population. Psoriasis vulgaris diagnoses were made and coded according to the 10th edition of the International Statistical Classification of Diseases and Related Health Problems. Age-specific and sex-specific incidences were calculated using data from 2009 to 2011. There was a rise in the age- and sex-specific incidences of psoriasis vulgaris through midlife, reaching a peak at the age of 60 and subsequently declining for both genders. The peak incidence for men, at 130 cases per 100,000 person-years, slightly exceeded the peak incidence for women of 117 per 100,000 person-years. An increase in the overall incidence rate can also be observed over the course of the three-year period covered by the data. Considerable variations in the age- and sex-specific incidences of psoriasis vulgaris can be seen across the lifespan. Nevertheless, the overall age-standardized incidence for the German population was low compared to other European countries.

**Supplementary Information:**

The online version contains supplementary material available at 10.1007/s00403-023-02796-y.

## Introduction

Psoriasis vulgaris is a common, chronic, and inflammatory condition that, while prominently manifested on the skin, is fundamentally a systemic disease [1–3]. The occurrence of the pathognomonic skin lesions often leads to stigmatization, which can significantly impact many patients’ quality of life [1]. Due to its relatively high prevalence and potential impairment of both work productivity and daily activity, psoriasis vulgaris has a high socioeconomic impact. The epidemiology of psoriasis vulgaris has been investigated in numerous small-scale studies in recent years, but the reliability of those findings is often limited due to the sample sizes or study designs. Experts have, therefore, consistently recommended large population-based studies to provide more reliable information on the age- and sex-specific distributions of psoriasis vulgaris [1–3]. The datasets in the current study included 65 million people with national statutory health insurance in Germany and were compiled within the framework of a morbidity-based risk adjustment. This study was to provide a comprehensive understanding of the age-specific and sex-specific incidence of psoriasis vulgaris in Germany. The findings of the study were compared with data from other national population-based studies.

## Methods

The study design was a retrospective, population-based, cross-sectional study using administrative health insurance data. The data were collected by Germany’s national statutory health insurance companies within the framework of morbidity-based risk adjustment—specifically, for the Morbidity-Based Risk Structure Compensation (morbiditätsorientierter Risikostrukturausgleich, Morbi-RSA) dataset [4–6]. The datasets were administered and were supplied by the German Institute of Medical Documentation and Information. According to the published German guideline, psoriasis vulgaris was defined and diagnosed by physicians using the definitions and codes from the 10th edition of the International Statistical Classification of Diseases and Related Health Problems [7, 8]. The data included all German residents who had statutory insurance from January 2009 to December 2012 (Supplementary Table 1). This study thus captured a substantial segment of the population (80%), representing around 65 million people. Residents with private health insurance and those without any insurance coverage were excluded from the study.

Unlike other studies, this research was not confined to a specific age group but spanned from birth to 100 years old, with sex-specific incidence helping to develop a picture of the distribution of psoriasis vulgaris through different life stages [3, 9]. The absolute numbers from the morbidity-based risk adjustment were used to calculate age- and sex-specific prevalences, and these data were published by Deike et al. (Supplementary Fig. 1) [10]. For the subsequent calculation of incidence, the method presented by Brinks et al. was employed, which incorporated prevalence, general mortality of the population, and the relative mortality associated with psoriasis vulgaris [11, 12]. Due to a lack of data on disease-associated mortality for the German population, a systematic literature search was conducted on this topic. The values for the relative mortality of psoriasis vulgaris were taken from a Danish national study [13], which we considered justifiable given that Denmark shares similar socioeconomic, demographic status, and lifestyle with Germany. The age-standardized incidence was then determined to enable comparison with studies from other countries.

## Results

### Study population

In 2009, the study population comprised 64,637,752 individuals, which had expanded to 65,792,296 by the end of the period covered by the data in 2012. Throughout the observation period, women participants slightly outnumbered men. The total number of psoriasis vulgaris patients grew from 1,419,537 in 2009 to 1,512,769 in 2012 (Supplementary Table 1).

### Age-specific and sex-specific incidence of psoriasis vulgaris

**Fig. 1:**
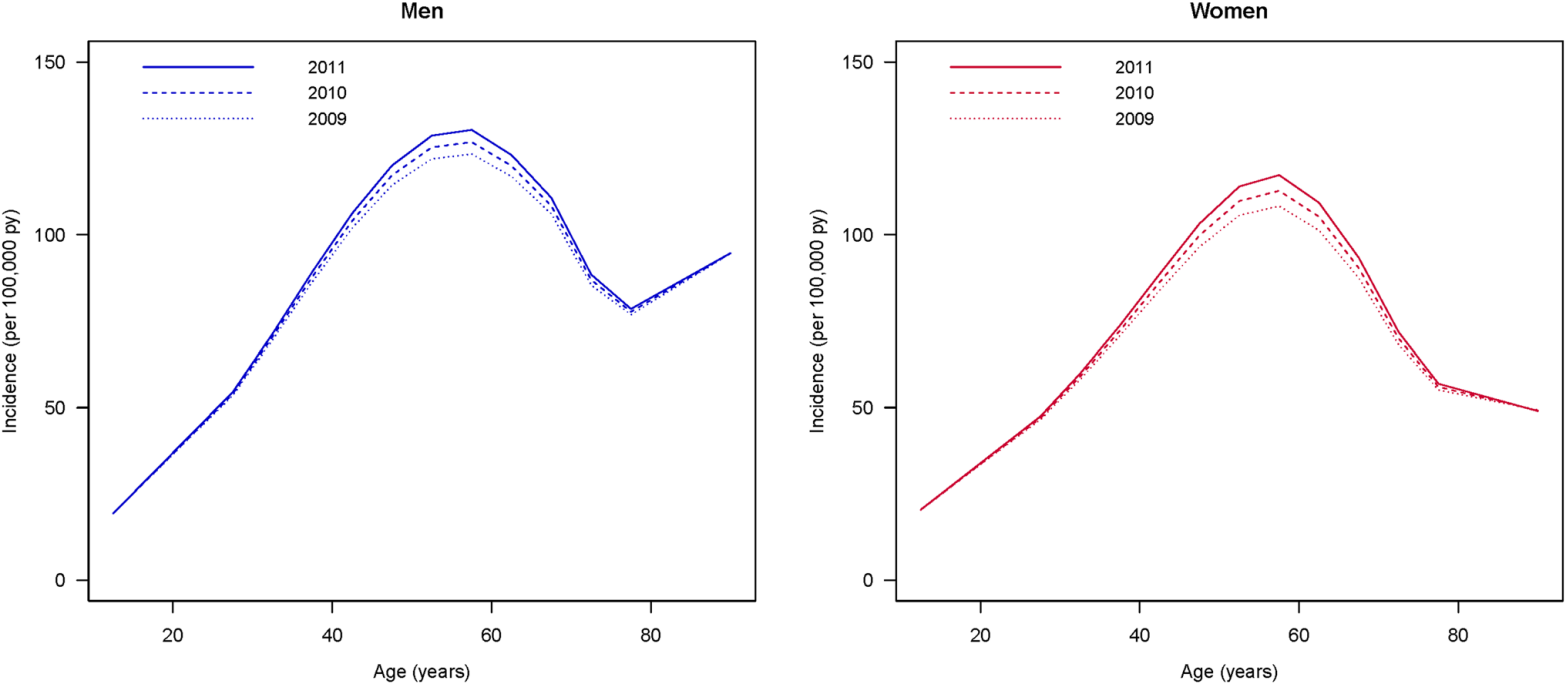
Age-specific and sex-specific incidence of psoriasis vulgaris (per 100,000 person-years) in Germany between 2009 and 2011

The age- and sex-specific incidences of psoriasis vulgaris rose until midlife, peaking at the age of 60: 130 cases per 100,000 person-years for men and 117 per 100,000 person-years for women (Fig. 1). After this peak, a steady decline was observed from the age of 60 onwards, but by the age of 80, we noted a divergence: while the incidence for women consistently declined, the incidence for men showed a modest increase. Despite these gender differences, a general upward trend in overall incidence was noted over the course of the period being studied.

### Age-standardized incidence of psoriasis vulgaris

For men, the age-standardized incidence increased from 77.97 cases per 100,000 person-years (95% confidence interval [CI] 75.62–80.30) in 2009 to 81.04 per 100,000 person-years (95% CI 78.47–83.59) in 2011. A similar upward trend was observed for women over the course of the period being studied. Specifically, the age-standardized incidence for women increased from 65.36 cases per 100,000 person-years (95% CI 63.19–67.55) in 2009 to 68.92 per 100,000 person-years (95% CI 66.48–71.39) in 2011.

## Discussion

This study offers a detailed age- and sex-specific picture of the incidence of psoriasis vulgaris, which has not previously been examined by the dermatology and rheumatology research community in Germany. As shown in Fig. 1, the majority of initial diagnoses are made before the age of 60, which suggests that the primary age of manifestation of psoriasis vulgaris is from early- to mid-adulthood. The incidence rate, which is strongly age specific, is consistent with studies by Egeberg et al. and Schonmann et al*.* [1, 14]. The study cohort also showed a disparity between the sex-specific incidences, with a slight predominance in men. This is inconsistent with a study in Canada by Eder et al. who found that both sexes were equally affected by psoriasis vulgaris [3], and our previously published study on the psoriatic arthritis subgroup using the same dataset, in which women appeared to outnumber men [10]. Nonetheless, due to the multifaceted origins of the disease, the exact cause of this age- and sex-specific pattern remains unclear.

The overall incidence in the current study rose consistently during the three-year period covered by the data (Fig. 1), which is consistent with the findings by Sewerin et al*.* who reported a similar upward trend in the prevalences of psoriasis [15]. The evolution and development of psoriasis vulgaris incidence over time remains a debated subject in the literature. A study by Icen et al. found rising incidence over time [16], while others have suggested either a steady or declining incidence rate [2, 3]. After considering the research methodologies of these studies, our incidence data estimations appear to provide more reliable information and more accurately reflect the actual situation in Germany. This is because our study data were sourced from the claims data of primary and secondary healthcare settings in Germany, which allowed registered physicians to diagnose psoriasis vulgaris with clinically verified codes, thus minimizing potential misclassification [15, 17, 18].

**Fig. 2:**
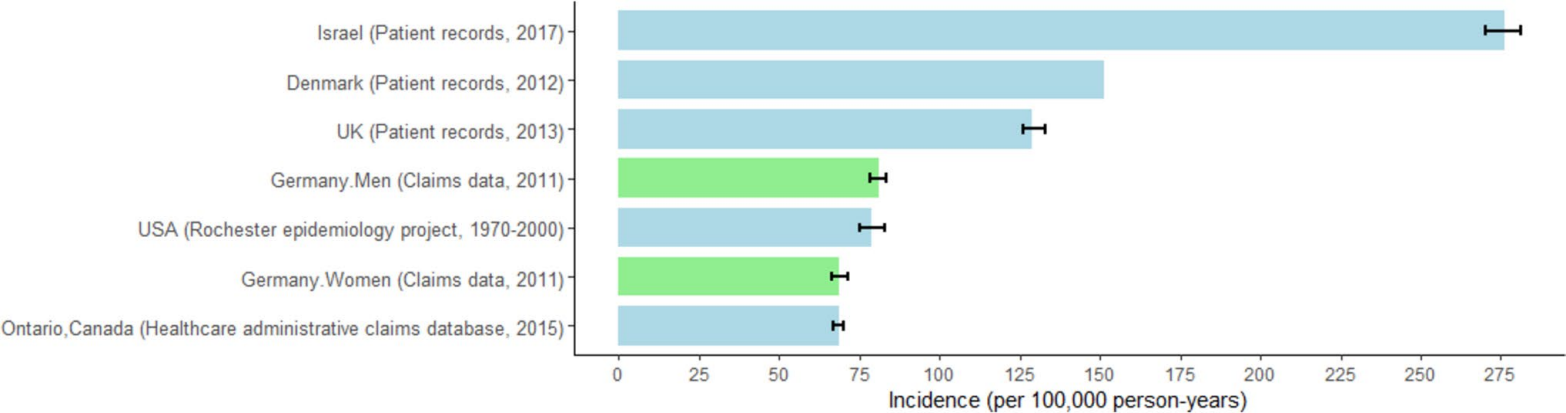
Global incidence of psoriasis vulgaris—Population-based incidence of psoriasis vulgaris in German. The data sources for the German study were retrieved from the national statutory health claims data. For the Danish study, data were sourced from all inpatient and outpatient (ambulatory) hospital consultation records in the Danish National Patient Register. The UK study used a comprehensive primary care database that maintains complete electronic patient records (including diagnoses, prescriptions, test results, and hospital referrals) from participating family practices across the U.K. The Canadian study extracted information from healthcare administrative claims database in Ontario, Canada, representing 40% of the Canadian population. The Israeli study used a continuously updated unified electronic medical file containing both administrative and clinical data. Lastly, the USA data were derived from the Rochester Epidemiology Project and from inpatient and outpatient records in Olmsted County, Minnesota, USA. All data presented in the graph from different countries were from population-based studies. We selected the data based on the most recent publication years. This selection not only offers a more contemporary representation of the incidence of psoriasis in these countries but also facilitates more accurate comparisons among these studies

We found that the age-standardized incidence of psoriasis vulgaris among the German population was 73 cases per 100,000 person-years, which was significantly lower than that reported in both the Danish and UK studies (Fig. 2) [1, 2]. Compared to a recent multinational systematic review, the incidences calculated in our study are comparatively low [19]. Nevertheless, despite disparities with the existing literature—including differences in study designs and cohort limitations—the current study has the advantage of a large sample comprising a majority of a nation’s population, which is quite exclusive in Europe. Furthermore, by including the population from birth to 100 years old, we can provide a comprehensive epidemiological overview on a nationwide scale.

For men, we observed a rise in incidence from the age of 80 (Fig. 1), which might be due to an overestimated disease-related mortality drawn from the Danish study [13]. The incidence likely has a strong interplay with the mortality rate ratio. German health experts would speculate that the future disease-related mortality in the German cohort might remain constant or slightly decline, considering current innovative medication and treatment strategies. This hypothesis could be verified through further studies with longer longitudinal cohorts. Interestingly, the incidence curve in our study deviated from the conventional rheumatology textbook depiction, which typically suggests a trend for psoriasis vulgaris with two peaks—the first around the age of 30 and the second around the age of 60 [14]. Previous studies have reported that early manifestations of psoriasis vulgaris tend to have a strong genetic component, whereas later manifestations of the disease are less likely to be linked to familial genetics and may instead be influenced by multifactorial environmental conditions [14, 19, 20]. However, the research from Canada is consistent with our findings from Germany, with neither of these studies showing an obvious bimodal age distribution in the incidence of psoriasis [3]. The reasons for this epidemiological discrepancy remain unclear, but one plausible explanation might be the data sources themselves: both our study and the Canadian study rely on claims data, while other studies have been based on clinically registered data from hospitals [3]. Furthermore, regional or environmental factors specific to Germany might result in disease onset being different than in other populations.

Finally, the age- and sex-specific frequency distributions of psoriasis vulgaris carry significant clinical and practical implications. Psoriatic arthritis—a condition associated with psoriasis that affects the joints—appears in a large fraction of psoriasis patients, with data indicating a high prevalence up to 30% among those with primary psoriasis [15, 21]. This suggests a potential progression to psoriatic arthritis among older people, who have a higher prevalence of psoriasis [10]. On the other hand, existing evidence shows that predicting the first onset of psoriatic arthritis in those already diagnosed with psoriasis is challenging [22]. The relationship between plaque psoriasis and joint complications is neither universally accepted nor fully understood in modern dermatology and rheumatology research. Consequently, determining the age- and sex-specific frequency distributions of psoriasis vulgaris is imperative for targeting treatment strategies in the context of psoriatic arthritis diagnosis. Furthermore, the existing literature emphasizes that severe psoriatic arthritis can lead to escalated comorbidities, resulting in a notably higher disease-related mortality, which can ultimately affect the incidence [13].

This study has a limitation in that we were only able to analyze incidence data from January 2009 to December 2011, primarily because we have not been granted access to newer data for at least the past five years. Furthermore, while we anticipate that applications for more recent data will be granted via the online Health Research Data Center (Forschungsdatenzentrum Gesundheit) in 2023, the new center is currently under construction, preventing any requests from being approved at this time [23]. Finally, there is an inherent delay in the data itself: death reports, as well as other medical diagnoses and treatment reports for some insurance holders, such as those residing abroad, can take up to three years to be recorded in the database, so even the most recently compiled data describes a period several years in the past.

In conclusion, this study fills a gap in the research on the epidemiology and population-based incidence of psoriasis vulgaris in Germany. Despite observing gender differences, a general upward trend in overall incidence was noted over the studied period. Our findings, therefore, underline the importance for policymakers of early detection, proactive screening, and secondary prevention measures for both psoriasis vulgaris and psoriatic arthritis.

### Supplementary Information

Below is the link to the electronic supplementary material.

Supplementary file1 (DOCX 64 KB)

## Data Availability

Due to the data protection laws in Germany, the datasets generated and/or analyzed during the current study cannot be made publicly available. The German law prohibits that the claims data affecting about 65 million people are used for other purposes than for research. Access to the data is granted to qualified research institutions upon request at the Forschungdatenzentrum (FDZ) in accordance with §§ 303a, 303f of Sozialgesetzbuch (SGB) V. Detailed information regarding the process for applying to obtain data access can be found on the website of the FDZ (in German) (https://www.forschungsdatenzentrum-gesundheit.de/das-fdz). Additionally, relevant laws pertaining to this matter are available on the website (in German) (https://www.gesetze-im-internet.de/sgb_5/BJNR024820988.html#BJNR024820988BJNG008700308).
