# Supplementary material for: Population-based incidence of psoriasis vulgaris in Germany: analysis of national statutory insurance data from 65 million population
Source: Arch Dermatol Res. 2024 Jan 4;316(2):65. doi: 10.1007/s00403-023-02796-y (PMC10766663; doi:10.1007/s00403-023-02796-y)
Supplement: Supplementary file 1 — Supplementary file1 (DOCX 64 KB) [file 403_2023_2796_MOESM1_ESM.docx]

**Title: Population-Based Incidence of Psoriasis Vulgaris in Germany: Analysis of National Statutory Insurance Data from 65 Million Population**

**Supplementary materials**

- **Supplementary Table 1 Characteristics of the study population**
- **Supplementary Figure 1 Age-specific and sex-specific prevalence of psoriasis vulgaris (per 1000) in Germany between 2009 and 2012**

**Supplementary Table 1 Characteristics of the study population**

| **Year** | **2009** | **2010** | **2011** | **2012** |
| --- | --- | --- | --- | --- |
| Population *(N)* | 64,637,752 | 63,962,071 | 64,988,016 | 65,792,296 |
| Proportion of Women *(%)* | 53.5 | 53.4 | 53.3 | 53.2 |
| Total number of psoriasis patients *(N)* | 1,419,537 | 1,440,807 | 1,477,333 | 1,512,769 |
| Total number of psoriatic arthritis patients *(N)* | 127,334 | 137,763 | 146,463 | 156,182 |

**Note:** Data collected for the morbidity-based risk adjustment of the statutory health insurance companies in Germany from 2009 to 2012.

**Note:** The data were published by Deike *et al* [1].

**Supplementary Figure 1 Age-specific and sex-specific prevalence of psoriasis vulgaris (per 1000) in Germany between 2009 and 2012**


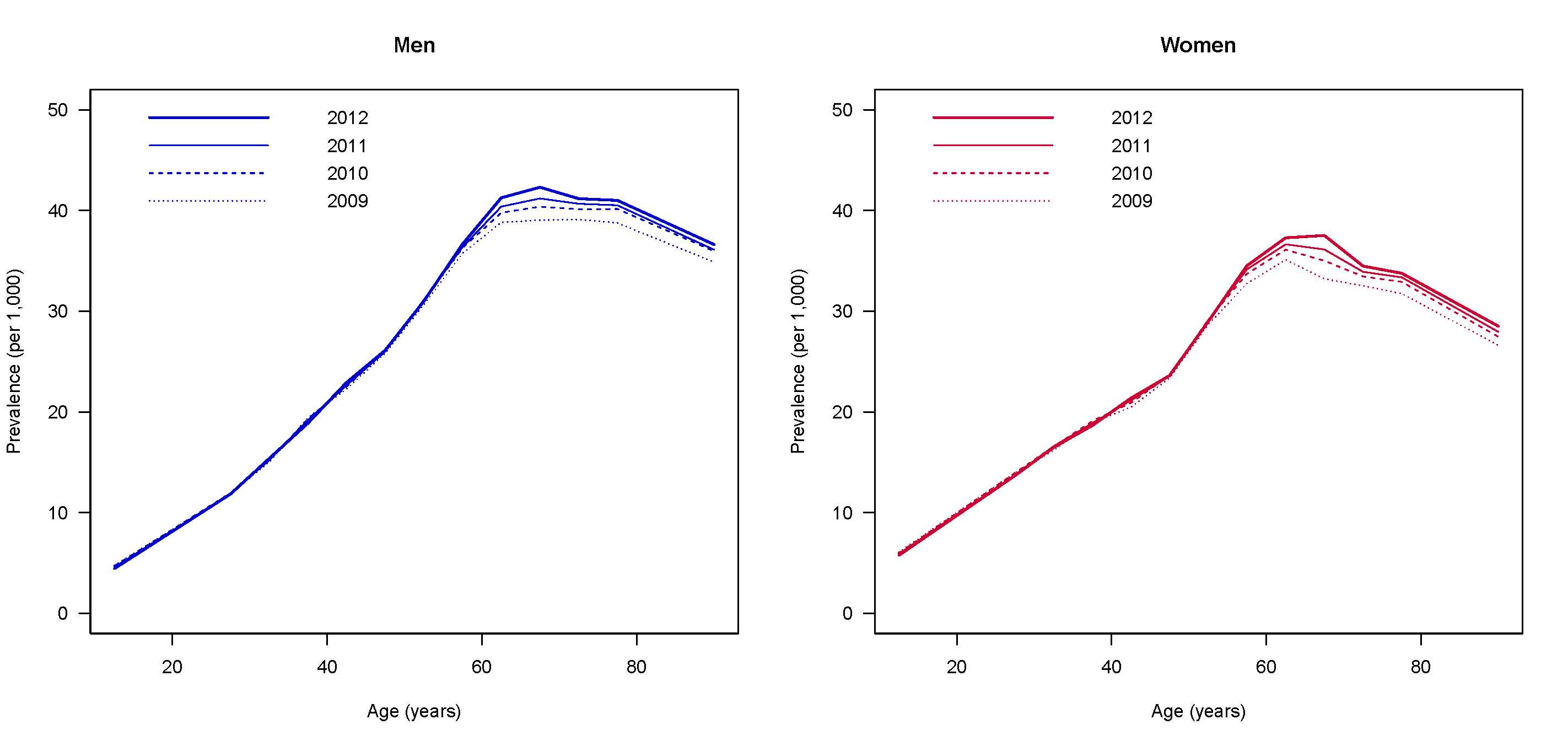


**Note:** The age- and sex-specific prevalences of psoriasis vulgaris increased consistently up to the sixth decade of life. After reaching a peak of approximately 40 per 1000 at this age, the rates plateaued or decreased only slightly. The prevalences for men were marginally higher than that for women.

**Note:** The data were published by Deike *et al* [1].

**REFERENCE**

1. Deike M, Brinks R, Meller S, Schneider M, Sewerin P (2021) Risk of psoriatic arthritis depending on age: analysis of data from 65 million people on statutory insurance in Germany. RMD Open 7(3). <https://doi.org/10.1136/rmdopen-2021-001975>
